# Evaluation of Prognostic Significance of Circulating Tumor Cells Detection in Rectal Cancer Patients Treated with Preoperative Radiotherapy: Prospectively Collected Material Data

**DOI:** 10.1155/2014/712827

**Published:** 2014-05-21

**Authors:** Dominika Nesteruk, Andrzej Rutkowski, Stanisław Fabisiewicz, Jacek Pawlak, Janusz A. Siedlecki, Anna Fabisiewicz

**Affiliations:** ^1^Department of Molecular and Translational Oncology, The Maria Skłodowska-Curie Cancer Center and Institute of Oncology, Roentgena 5, 02-781 Warsaw, Poland; ^2^Department of Medical Genetics, Institute of Mother and Child, 01-211 Warsaw, Poland; ^3^Department of Oncological Gastroenterology, The Maria Skłodowska-Curie Cancer Center and Institute of Oncology, 02-781 Warsaw, Poland; ^4^Department of Surgery, John Paul Western Hospital, 05-825 Grodzisk Mazowiecki, Poland

## Abstract

The aim of this study was to evaluate the prognostic value of circulating tumor cells (CTC) in nonmetastatic rectal cancer patients treated with short-term preoperative radiotherapy. In this single-center trial, 162 patients with rectal cancer after preoperative short-term radiotherapy (5 × 5 Gy) were recruited from January, 2008 to September, 2011. Clearance of CTC was determined in 91 patients enrolled in the molecular analysis. CTC presence was evaluated with real-time reverse transcription polymerase chain reaction assay (qPCR) based on the expression of three tumor genetic markers: carcinoembryonic antigen (CEA), cytokeratin 20 (CK20), and/or cancer stem cells marker CD133 (CEA/CK20/CD133). We found that CTC detection 7 days after surgery was of prognostic significance for the local recurrence (*P* value = 0.006). CTC detected preoperatively and 24 hours after resection had no prognostic value in cancer recurrence; however, there was a significant relationship between CTC prevalence 24 hours after surgery and lymph node metastasis (pN1-2). We also confirmed a significant clearance of CTC in peripheral blood (PB) 24 hours after surgery. Preoperative sampling is not significant for prognosis in rectal cancer patients treated with short-term radiotherapy. Detection of CTC in PB 7 days after surgery is an independent factor predicting local recurrence in this group of patients.

## 1. Introduction

Circulating tumor cells (CTC) can be detected in peripheral blood (PB) of cancer patients who have no evidence of overt metastases [1, 2]. Dissemination of tumor cells is therefore thought to occur early on in the cancer development. The presence of CTC in PB has proven to be of prognostic significance in patients with metastatic colorectal cancer [3, 4]. For nonmetastatic colorectal cancer, clinical significance of CTC is being investigated. Five studies have found that the presence of CTC postoperatively predicts poor disease-free survival, and in two studies, preoperative CTC predicted early recurrence and poor disease-free survival [5]. The review by Peach et al. [6] summarized that the presence of CTC in PB at least 24 h after tumor resection was an independent prognostic marker of recurrence, but there was no significant correlation between CTC and survival perioperatively. Additionally, significant differences in CTC detection rates in nonmetastatic cancer patients were observed [7] and the presence of CTC in nonmetastatic colon cancer was barely detectable with the CellSearch System—the only system approved for clinical routine use [8].

The identification of new markers for better patients risk stratification is of important clinical significance. Adjuvant chemotherapy has been shown to significantly improve outcomes of nonmetastatic stage III patients, but for stage II (node-negative) patients the benefit of this therapy is still discussed. The early tumor dissemination measured by CTC presence in stage II patients may be indicative of the application of adjuvant chemotherapy. Thus, monitoring of CTC in nonmetastatic cancer may represent a valuable marker of early spread of the disease in patients without overt metastases.

Among several studies only few focused on rectal cancer patients who received preoperative radiotherapy. Short-term 5 × 5 Gy preoperative radiotherapy apart from tumor reduction reduced local recurrence rates and improved overall survival compared with surgery alone [9]. Following radiotherapy, CTC have a trend to decrease [10]. Therefore determination of the prognostic value of CTC after radiotherapy in these patients is of importance and was a subject of our studies.

Given that the detection of CTC in nonmetastatic colon cancer with the CellSearch is inadequate [8], we decided to use real-time reverse transcription polymerase chain reaction assay (qPCR) previously developed by Iinuma et al. [11]. This multimarker assay is based on the expression of three genetic markers: carcinoembryonic antigen (CEA), cytokeratin 20 (CK20), and/or cancer stem cells marker CD133 (CEA/CK/CD133) and was shown to be a useful tool for evaluation of CTC as a prognostic factor in PB of colorectal cancer patients [11].

The aim of this study was to clarify the prognostic significance of CTC presence in PB after resection of nonmetastatic rectal cancer in patients treated with preoperative radiotherapy. We focused on the presence of CTC in samples taken preoperatively, 24 h, and 7 days after surgery.

## 2. Materials and Methods

### 2.1. Study Design

We performed our studies on 162 patients with rectal cancer after preoperative short-term radiotherapy recruited from January, 2008 to September, 2011, for trial examining the role of gentamicin collagen implant (GCI) in the risk of cancer recurrence. The local ethics committee at the Center of Oncology in Warsaw approved the study.

Participation in the study was open to patients with histopathologically confirmed adenocarcinoma of the rectum (cT3-4, N0-2, M0), located up to 12 cm from anal verge.

For a need of a previous study (unpublished) patients were randomized to two groups: one who received GCI implanted in the space created after mesorectal resection and control group without GCI. However, statistical analysis showed lack of influence of GCI implant on the results (data not shown); therefore, both groups were treated as one in the present study.

### 2.2. Healthy Control Group

Thirty-five patients without cancer from John Paul Western Hospital, Grodzisk Mazowiecki were enrolled into the study to determine cut-off value of the tumor markers expression in their blood samples.

### 2.3. Cell Lines

The human colorectal carcinoma cell lines T84 and COLO 205, obtained from the LCG Standards (Teddington, UK), were incubated in RPMI-1640 medium (Invitrogen-Gibco, Carlsbad, CA, USA) containing 10% fetal calf serum (Euroclone-Celbio, Pero, MI), 10 mM Hepes, and 1 mM sodium pyruvate (Sigma-Aldrich, St. Louis, MO), at 37°C in 5% CO_2_. According to the manufacturer's datasheet, cells express the surface glycoproteins carcinoembryonic antigen (CEA).

### 2.4. Sample Collection, RNA Extraction, and cDNA Synthesis

A 2.5 mL aliquot of peripheral blood from each patient was collected to PAXgene Blood RNA Tube (QIAGEN BD Company, NJ, USA) at three different time points: prior to resection, 24 h after surgery, and 7 days after resection. Tubes were stabilized in room temperature and frozen at −70°C until RNA extraction. Total RNA was isolated from samples with PAXgene Blood RNA Kit. The integrity of the RNA was established by electrophoresis on agarose precast gel (FlashGel System, Lonza Rockland, ME, USA) and RNA was quantified on NanoDrop spectrophotometer (Eppendorf).

500 ng of total RNA was reverse-transcribed using random primers and MultiScribe Reverse Transcriptase (High-Capacity cDNA Reverse Transcription Kit, Applied Biosystems, Foster City, CA, USA). The reaction mixture was incubated for 10 min at 25°C, then at 37°C for 120 min and for 5 min at 85°C. The quality of cDNA was assessed by the amplification of one of the housekeeping genes: glyceraldehyde 3-phosphate dehydrogenase (GAPDH) or DNA polymerase *β*(*polβ*) in the PCR reaction [12]. Housekeeping genes are expressed constitutively and therefore they reflect the total amount of cDNA in the sample. cDNA was stored at −20°C until use.

### 2.5. Real-Time Quantitative Polymerase Chain Reaction (qPCR)

Previous studies by Nakamura et al. [13] and Iinuma et al. [11] have shown that expression of CEA/CK20/CD133 mRNA was a useful tool for the detection of CTC in PB of colorectal cancer patients. Based on these studies, we measured the transcriptional levels of three genes: carcinoembryonic antigen [*CEA*], cytokeratin-20 [*CK20*], and prominin-1 [*CD133*] by means of qPCR using the relative quantification method (ΔΔCt method) [14]. Three housekeeping genes: PPIA, SDHA, and HPRT1 were used as reference genes to normalize levels of target genes to the amount of mRNA present in each sample as recommended by Vandesompele et al. [15]. Housekeeping genes were chosen based on the highest expression stability in experimental conditions run on the mRNA matrixes isolated from various biologic materials (peripheral blood (PB) of colon tumor patients, PB from healthy volunteers, cell lines T84 and COLO 201, and primary tumor tissue). The qPCR method was validated by the determination of the amplification efficiency of all target and housekeeping genes. Analysis of the slopes of standard curves created for dilution series of input T84 cell lines cDNA confirmed comparable high efficiencies and linear amplification. RNA extracted from T84 and COLO 205 cell lines were used in test experiments for method optimization and in the final experiments as a positive control.

Due to a low expression of the marker genes and high Ct values obtained in the test experiments, a preamplification (PreAmp) step was added. Preamplification enhances the sensitivity of real-time RT-PCR, especially for low abundance genes and can establish substantially higher cDNA amounts, which in turn allows to increase the number of analyzable target genes in one sample.

Since initial experiments comparing the 25 *μ*L and 5 *μ*L volume of PreAmp reaction showed identical PreAmp results, we used 5 *μ*L volume further on due to cost-effectiveness. The PreAmp reaction conditions involved the amplification of 1.25 *μ*L of 5 times diluted cDNA in a 5 *μ*L reaction consisting of 3.75 *μ*L TaqMan PreAmp Master Mix and pooled primer/assay mix (0.2x, each assay). PreAmp reaction was carried out with the following program: denaturation at 95°C for 10 min and 14 cycles of amplification (15 sec at 95°C, 4 min at 60°C). The preamplified products were then diluted at a ratio of 1 : 100 and were used as templates for the qPCR analysis.

TaqMan Gene Expression Assays specific for* CEA*,* CK20*, and* CD133*, recommended by the company as “best coverage”, were purchased from Applied Biosystems. The qPCR assay was performed using the ABI PRISM 7900 HT Sequence Detection System. The PCR reaction proceeded in a mixture (4 *μ*L) containing 2 *μ*L of 2x TaqMan Gene Expression PCR Master Mix, 0.2 *μ*L of 20x TaqMan Gene Expression assay (all reagents from Applied Biosystems), and 1.8 *μ*L of cDNA template. Forty-five cycles of amplification were performed at 95°C (15 seconds) and 60°C (1 minute).

Results from qPCR reactions of studied genes were normalized to the Ct values of reference genes and converted to the fold-change (Fc) values, relative to the calibrator sample [16]. The calibrator sample was the sample with the lowest ΔCt value in the testing group.

### 2.6. Statistical Analysis

Statistical analysis was conducted in the STATISTICA program (StatSoft, PL). Results were considered statistically significant at *P* value < 0.05. Two-sided *P* value was used. Fc values equal to 0 (no change in relative expression level) were excluded from analysis.

On the basis of marker genes expression in healthy controls (*n* = 27 for postoperative samplings, *n* = 8 for preoperative sampling), the cut-off value of tumor marker was defined as the one with the highest Fc. One of the Fcs of CEA, CK20, and/or CD133 that exceeded cut-off value in patient group indicated the presence of the CTC (CTC (+)), as defined previously in studies by Iinuma [13, 17]. Fc below cut-off value denoted lack of CTC (CTC (−)). Pearson's chi-square test was used to assess differences in clinical factors between patients with and without CTC presence in each blood sampling.

## 3. Results

Among 162 patients, 113 (69.8%) had complete blood sampling and were enrolled to the molecular analysis. Samples of 19 (16.8%) patients were excluded from further analysis due to the lack of RNA integrity. In the final experiments of molecular analysis there were 91 patients (36 received GCI). No significant differences in the CTC presence were observed between patients who received GCI implant and patients in the control group in either of the blood sampling (data not shown); therefore, all patients were analyzed as one group.

### 3.1. Patient Characteristics

In total, 60 men (66%) and 31 women (34%) with a mean age of 67 years were analyzed. 41 patients had lymph nodes metastases in pathologic evaluation. One patient after preoperative radiotherapy treatment had a total remission of cancer (Table 1). A 2-year follow-up data is complete for all except one patient. Data concerning 3-year follow-up are currently available for 60 (65%) patients.

### 3.2. Circulating Tumor Cells Detection and Its Correlation with Clinicopathological Factors

The CTC were detected in 39 patients (43%) in preoperative blood samples, in 11 (12%) at 24 hours after surgery, and in 12 (13%) 7 days after surgery (Table 2).

Detectable preoperative CTC level did not reflect the lymph node metastasis status (pN0 versus pN1-2). On the contrary, at 24 hours after surgery, majority of patients positive for CTC presence (73%) had pN1-2 category (*P* value = 0.049). Similar relationship of CTC presence was found at the same sampling time with advanced invasion of tumor (pT3-4); however, it did not reach statistical significance.

In both preoperative and subsequent (24 h after) blood samples, the CTC detection rate increased with the disease severity (according to the TNM stage classification) but only in a sampling taken 24h after surgery this correlation was close to the statistical significance (*P* value = 0.07). This observation was not found at 7 days after surgery.

### 3.3. Prognostic Significance of Circulating Tumor Cells in Peripheral Blood

Prognostic significance of CTC was evaluated in analysis of tumor cells detection and disease recurrence (local recurrence and distant metastasis) or disease-related death events (Table 2). Seventeen patients (19%) had a disease recurrence, among them 6 had a local recurrence and 14 distant metastases; eight patients (9%) died.

We found an interesting correlation of CTC positivity with the local recurrence events (25% of CTC (+) patients versus 4% of CTC (−) patients; *P* value = 0.006) 7 days after surgery. No correlation of CTC prevalence with disease recurrence or cancer-related death was found in preoperative and at 24 hours after surgery blood sampling (Table 2). Disease-free survival analysis with Kaplan-Meier method will be possible to calculate when a 3-year follow-up for all patients will be available.

### 3.4. Clearance of Circulating Tumor Cells at 24 Hours after Surgery

**Figure 1:**
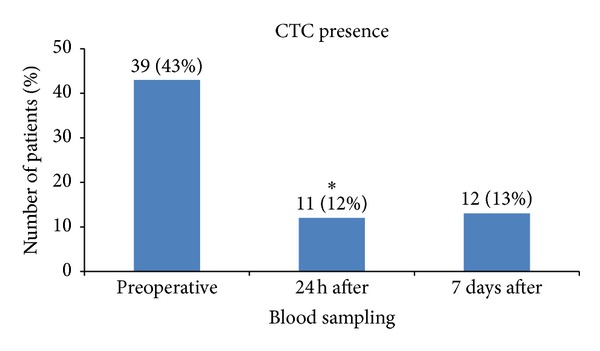
Circulating tumor cells prevalence in peripheral blood of rectal cancer patients after preoperative radiotherapy at three different time points: preoperative, 24 hours after tumor resection (24 h after), and 7 days after surgery (7 days after). *Statistically significant clearance of CTC at 24 hours after surgery (*P* value < 0.001).

There was a statistically significant (*P* value < 0.001) clearance of tumor cells in peripheral blood 24 hours after surgery, shown by 72% reduction in CTC positive patients in comparison with preoperative data (Figure 1). The CTC positive patients' percentage remained on the same level in the subsequent time point.

## 4. Discussion

The clinical significance of the detection of CTC in low stages of nonmetastatic rectal cancer in patients treated with short-term preoperative radiotherapy is so far unknown (not determined). Most studies focus on metastatic colorectal cancer where the presence of CTC in PB has proven to be of prognostic significance relating to progression-free survival (PFS) and overall survival (OS). Less data concerning clinical value of CTC is available for nonmetastatic colorectal cancer. Five studies performed with RT-PCR method have found that the presence of CTC postoperatively predict poor disease-free survival [5]. Only in two studies were CTC evaluated for clinical significance in preoperative samples. The one by Iinuma et al. [11] performed with RT-PCR reported that OS and DFS of patients with detectable CEA/CK/CD133^+^ mRNA were significantly worse than those of patients whose blood was negative for these markers. Importantly, the assay was prognostic only for patients in higher cancer stages: Dukes B and Dukes C but not prognostic for patients in Dukes' stage A or stage B who had favorable prognoses determined by the basis of clinical and pathologic features.

To evaluate and compare the presence of the CTC in preoperative and postoperative samples, we chose a multimarker test previously developed by Iinuma et al. [11]. The multimarker test provides better sensitivity than the one marker test since it takes into account phenotypic heterogeneity of tumor cells populations. Tumor cells disseminated into the bloodstream may be at various levels of differentiation and therefore may lose the expression of epithelial markers. The use of additional stem cells marker CD133+ allows detecting not only the CK20+ and CEA+ subpopulation of the CTC but also the CD133+ subpopulation—more aggressive and resistant to apoptosis and therefore having great potential for metastases. Additionally, qPCR method allows eliminating the false-positive results from healthy individuals by the determination of the cut-off point and eliminating the differences in the amount of cDNA template between samples by normalization to the reference genes.

In our study, the percentage of CTC-positive patients ranged from 11% to 50% in different blood samples. This is consistent with the results obtained for colorectal cancer by Iinuma et al. and other groups [11, 18]. Changes in the level of the CTC in subsequent samples corresponded to the previously published results [6, 19], indicating a proper cut-off determination.

Analysis of our results showed lack of clinical significance of CTC detection in samples taken preoperatively. No statistically significant differences in local recurrence and metastases were detected between patients with CTC presence and absence in peripheral blood. Additionally, CTC detected preoperatively were not prognostic according to TNM.

The presence of CTC in postoperative blood samples was shown to be statistically significant only with regard to local recurrence. This effect was noticed only in samples taken 7 days after surgery. The correlation of CTC positivity with the local recurrence 7 days after surgery may be informative for clinicians; however, this result should be confirmed in further studies.

We also observed some trends in samples taken 24 h after surgery. Detectable CTC level reflected metastasis to lymph nodes (pN1-2) with statistical significance (*P* value = 0.049). CTC presence correlated with TNM stage as shown previously [17, 20, 21]; however, it did not reach statistical significance. These results indicate that CTC positivity in our patients treated with preoperative radiotherapy reflects stage of the disease rather than prognosis.

During the rectal cancer resection, the surgeon paradoxically contributes to the spread of the primary tumor cells. Removal of rectal adenocarcinoma located below the peritoneal wave requires that the surgeon manually presses the tumor before the ligation of all paths of blood and lymph drainage from the rectum. This procedure leads to a release (leaking) of tumor cells that easily invade the bloodstream through the extensive wound which has a good blood supply. Many publications confirmed elevated levels of the CTC in intraoperative blood samples [6]. The highest clearance of the CTC was noticed within 24 hours after surgery [19] as a result of their elimination by the host's immune system. The same observation is concluded from current study. Therefore, we consider blood samples taken 24 hours after surgery to be the most informative. The CTC prevalence in pN1-2 group was statistically significantly higher than in pN0 group which may additionally emphasize the importance of a 24 h time point. Furthermore, only for this blood sampling time, we observed a trend of correlation of the CTC presence with TNM tumor stage (*P* value = 0.07).

However, it should be stressed that not every cancer cell that is shed from the tumor into circulation contributes to metastases; less than 0.1% of the CTC survive in the bloodstream, and only 0.01% are able to form metastases. Furthermore, the level of expression of molecular markers does not reflect the exact number of the CTC in patient's sample, only their higher or lower level. Differences in the expression of molecular markers reflect differences in the number of CTC. However, it cannot be excluded that these differences are also the evidence of variation in expression within the non-homogeneous population of cancer cells in the single blood sample.

Some studies question the prognostic value of the CTC in metastasis taking into account a small percentage of the CTC capable of forming metastases and low expression of molecular markers in these cells [22]. There are several limitations in the determination of the CTC in the blood. One explanation of detection failure is that circulating cells are not homogeneously distributed and noncontinuously shed into circulation [23, 24]. According to this assumption, the concentration of CTC in the blood is not constant, the spreading takes place at intervals in time [25], and therefore the detection of CTC in single blood collection may be hampered. Additionally, preoperative chemoradiation may decrease the rate of rectal cancer cells in the blood and bone marrow as shown by Kienle et al. [26] which may further affect detection of these cells after radiotherapy.

Thorough analysis of the effect of CTC detection on distant metastasis in patients after radiotherapy requires at least a 3-year follow-up. To date, the 3-year follow-up is available for only 60 out of 91 analyzed patients. This analysis will be possible to perform in 2 years.

## 5. Conclusions

We found that preoperative sampling is not significant for prognosis in rectal cancer patients treated with short-term radiotherapy. Detection of CTC in peripheral blood 7 days after curative surgery is an independent factor predicting local recurrence in this group of patients. To our best knowledge, this is the first study of the evaluation of prognostic significance of CTC performed on a homogenous group of patients who all received short-term preoperative radiotherapy. The evidence presented in this study provides a basis for future multicenter trials with a larger number of patients.

## Figures and Tables

**Table 1:** Patient characteristics.

	Patients
	*n* = 91
Sex	
Male	60
Female	31
Age (years): mean ± SD	66 ± 11.5
WHO status*	
0	34
1	57
pT	
0	1
1	0
2	28
3	61
4	1
pN	
0	50
1	26
2	15
TNM	
I	23
II	26
III	41
Cancer remission	1

*World Health Organization performance status.

**Table 2:** Correlation of circulating tumor cells (CTC) detection in peripheral blood with clinicopathological factors and disease recurrence in patients with a radical resection of primary rectal cancer after preoperative radiotherapy.

	Total patients *n* = 91	Preoperative		24 hours after		7 days after	
		CTC (+) *n* = 39	*P* value	CTC (+) *n* = 11	*P* value	CTC (+) *n* = 12	*P* value
pN0	50	20 (51%)	0.54	3 (27%)	**0.049**	8 (67%)	0.38
pN1-2	41	19 (49%)		8 (73%)		4 (33%)	

pT0–2	29	11 (28%)	0.52	1 (9%)	**0.08**	5 (42%)	0.43
pT3-4	62	28 (72%)		10 (91%)		7 (58%)	

TNM I	23	8 (20%)	0.63	0	**0.07**	3 (25%)	0.54
TNM II	26	12 (31%)		3 (27%)		5 (42%)	
TNM III	41	19 (49%)		8 (73%)		4 (33%)	

Cancer recurrence	17	7 (18%)	0.88	2 (18%)	0.88	3 (25%)	0.55
Local recurrence	6	3 (8%)	0.71	1 (9%)	0.72	3 (25%)	**0.006**
Metastasis	14	6 (15%)	1.00	2 (18%)	0.78	1 (8%)	0.47

Cancer related death	8	4 (10%)	0.67	1 (9%)	0.97	0	0.25

CTC (+): number of patients with circulating tumor cells; pN: lymph nodes metastasis; pT: depth of tumor invasion.
